# Effect of total knee replacement on skeletal muscle mass measurements using dual energy X-ray absorptiometry

**DOI:** 10.1038/s41598-023-29069-y

**Published:** 2023-02-19

**Authors:** Jae Young Jang, Miji Kim, Daehyun Lee, Chang Won Won

**Affiliations:** 1 Department of Biomedical Science and Technology, Graduate School, Kyung Hee University, Seoul, 02447 South Korea; 2 Department of Biomedical Science and Technology, College of Medicine, East-West Medical Research Institute, Kyung Hee University, Seoul, 02447 South Korea; 3 Department of Family Medicine, Elderly Frailty Research Center, College of Medicine, Kyung Hee University, 23, Kyung Hee Dae-Ro, Dongdaemun-Gu, Seoul, 02447 South Korea

**Keywords:** Diagnosis, Geriatrics

## Abstract

Sarcopenia is becoming prevalent in an increasing number of older adults undergoing total knee replacement (TKR) surgery. Metal implants may overestimate lean mass (LM) measured using dual-energy X-ray absorptiometry (DXA). This study aimed to examine the effects of TKR on LM measurements according to automatic metal detection (AMD) processing. The participants from Korean Frailty and Aging Cohort Study, who had underwent TKR were enrolled. A total of 24 older adults (mean age 76.4 ± 4.0 years, 92% female) were included in the analysis. The SMI with AMD processing was 6.1 ± 0.6 kg/m^2^, which was lower than that without AMD processing of 6.5 ± 0.6 kg/m^2^ (*p* < 0.001). The LM of the right leg with AMD processing was lower than that without AMD in 20 participants who had underwent TKR surgery on the right (5.5 ± 0.2 kg vs. 6.0 ± 0.2 kg, *p* < 0.001), and that of the left leg was also lower in with AMD processing than in without AMD processing in 18 participants who had underwent TKR surgery on the left (5.7 ± 0.2 kg vs. 5.2 ± 0.2 kg, *p* < 0.001). Only one participant was classified as having low muscle mass without AMD processing, but this came to four after AMD processing. LM assessment in individuals who had TKR could be significantly different according to the use of AMD.

## Introduction

Sarcopenia is defined as the progressive loss of muscle mass, muscle strength, and/or physical performance that occurs with aging^1^. Various working groups have developed consensus guidelines for the diagnosis of sarcopenia, and these guidelines recommend measuring appendicular lean mass (ALM) using dual-energy X-ray absorptiometry (DXA)^2–5^. Therefore, the accurate measurement of lean mass (LM) is an important factor in diagnosing sarcopenia.

DXA is a noninvasive tool for measuring body composition aspects, such as bone mineral content (BMC), LM, and fat mass (FM)^6^ with high accuracy, with good accessibility, and at a reasonable price^7^. High-density objects, such as metal rods, can affect the imaging area while measuring body composition using DXA^6^. A previous study reported that hip arthroplasty metals underestimated FM and overestimated LM of the side of hip replacement compared with that of the non-replacement side^8^. Although the gold standard for measuring LM in individuals with metal implants has not yet been established, the most common solution is to exclude those with metal implants from the analysis^7,8^ or to use automatic metal detection (AMD) software provided by the manufacturer to remove metal implants in the analysis. Also, a few studies have estimated ALM by substituting LM of the leg with a metal implant with that of the other leg without the metal implant^9,10^.

We searched for studies published from January 2010 to August 2021 in the PubMed search engine using the following keywords: “sarcopenia” AND “dual energy X-ray absorptiometry”. Our inclusion criteria for the search were as follows: (1) population aged > 60 years, (2) DXA use for body composition analysis, and (3) LM as the study outcome. The selected studies were classified according to whether they considered metal implants while presenting ALM, specifically mentioning metal implants, prosthesis, arthroplasty, and joint replacement (Table S1). We identified 595 studies that met our inclusion criteria. Of the 595 studies, only 78 (13.1%) considered metal implants in the study participants: 74 studies excluded participants with metal implants, two studies used AMD software^11,12^, and two studies estimated ALM by replacing the LM of the leg with a metal implant with that of the other leg without the metal implant (Table S1)^9,10^. The remaining 517 studies (86.9%) did not report whether their participants had metal implants or not.

The demand for total knee replacement (TKR) surgery is dramatically increasing due to the growing prevalence of knee osteoarthritis (OA) which can cause adverse effects on LM^13,14^. In the United States, a total of 7.8 million primary TKRs were performed, and the number of surgeries increased by 224% from 1993 to 2012^15^. In Germany, the number of TKR surgeries increased by 33% from 2005 to 2016^16^. In South Korea, about 2 million people (4% of the total South Korean population) undergo TKRs annually, and the number of TKR surgeries per year is rapidly increasing^17^. Sarcopenia among patients with end stage OA of the knee is not uncommon^13^. According to the 2016–2017 baseline survey of Korean Frailty and Aging Cohort Study (KFACS), the prevalence of TKRs and total hip replacements (THRs) in community-dwelling older adults is 8.8% (2.7% in men and 14.1% in women) and 2.5% (1.8% in men and 2.5% in women), respectively. Furthermore, granted that older adults account for approximately 80% of total TKR surgeries^18^, considering TKR metal implants while using DXA in older adults is important. However, there has been no study to investigate the effect of TKR on LM measurement on the TKR side leg.

Therefore, the purpose of this study was to investigate the effect of TKR on LM measurements using DXA according to AMD processing and the substitution protocol (doubling of LM on the non-TKR side).

## Methods

### Study participants

The KFACS is a nationwide, multicenter, longitudinal cohort study conducted at 10 centers (eight university hospitals and two public health centers) in rural and urban areas across the country, and the baseline survey was conducted in 2016–2017^19^. The sample consisted of participants aged ≥ 70–84 years who were independently mobile, and participants were recruited from age- and sex-stratified community residents at 10 centers. The details of the study design have been described previously^20^. There were 3,014 participants in the baseline survey, and 2403 of them underwent DXA measurements at eight university hospital centers (Lunar, GE Healthcare, Madison, WI; Hologic DXA, Hologic Inc., Bedford, MA). Of the eight centers, Kyung Hee University Hospital recruited 303 participants for the baseline survey and had their measurements taken using GE Lunar iDXA; out of them, 61 participants were found to have orthopedic surgical implants: 17 had lumbar pins, 2 had pins in the left upper arm, 5 had right hip joint pins, 24 underwent TKR surgery, and 13 had multiple implants. The 24 participants who had underwent TKR surgery were selected for the study: six had right-knee TKR, four had left-knee TKR, and 14 underwent TKR surgery in both knees. As this study was started as a pilot study, the sample size for our study was determined based on previous studies that compared body composition measured by DXA with number varying from 13 to 21^6,8,21–23^. The Clinical Research Ethics Committee of Kyung Hee University Hospital approved the KFACS protocol (Institutional Review Board number: 2015-12-103). This study did not require approval by the Institutional Review Board of the Clinical Research Ethics Committee of the Kyung Hee University Medical Center (Institutional Review Board number: 2022-04-039). All the participants gave their informed written consent and all methods were performed in accordance with the relevant guidelines and regulations (the Declaration of Helsinki).

### Measurement of body composition

Whole-body composition and ALM were measured using DXA (Lunar iDXA; GE Healthcare, Madison, WI, USA; enCORE™ 2007 VERSION 11.40.004). The participants were positioned according to the manufacturer’s protocols and were asked to remove all metal accessories before scanning. The participants laid down on the scanner table with their limbs close to their bodies. The arms, legs, and trunk segments were divided manually based on anatomical landmarks using the DXA analysis software. One specialized DXA technician identified the bones and fat tissues, and ALM was calculated as the sum of the LMs of the limbs. Skeletal muscle mass index (SMI) was calculated using the formula ASM/height^2^.

**Figure 1:**
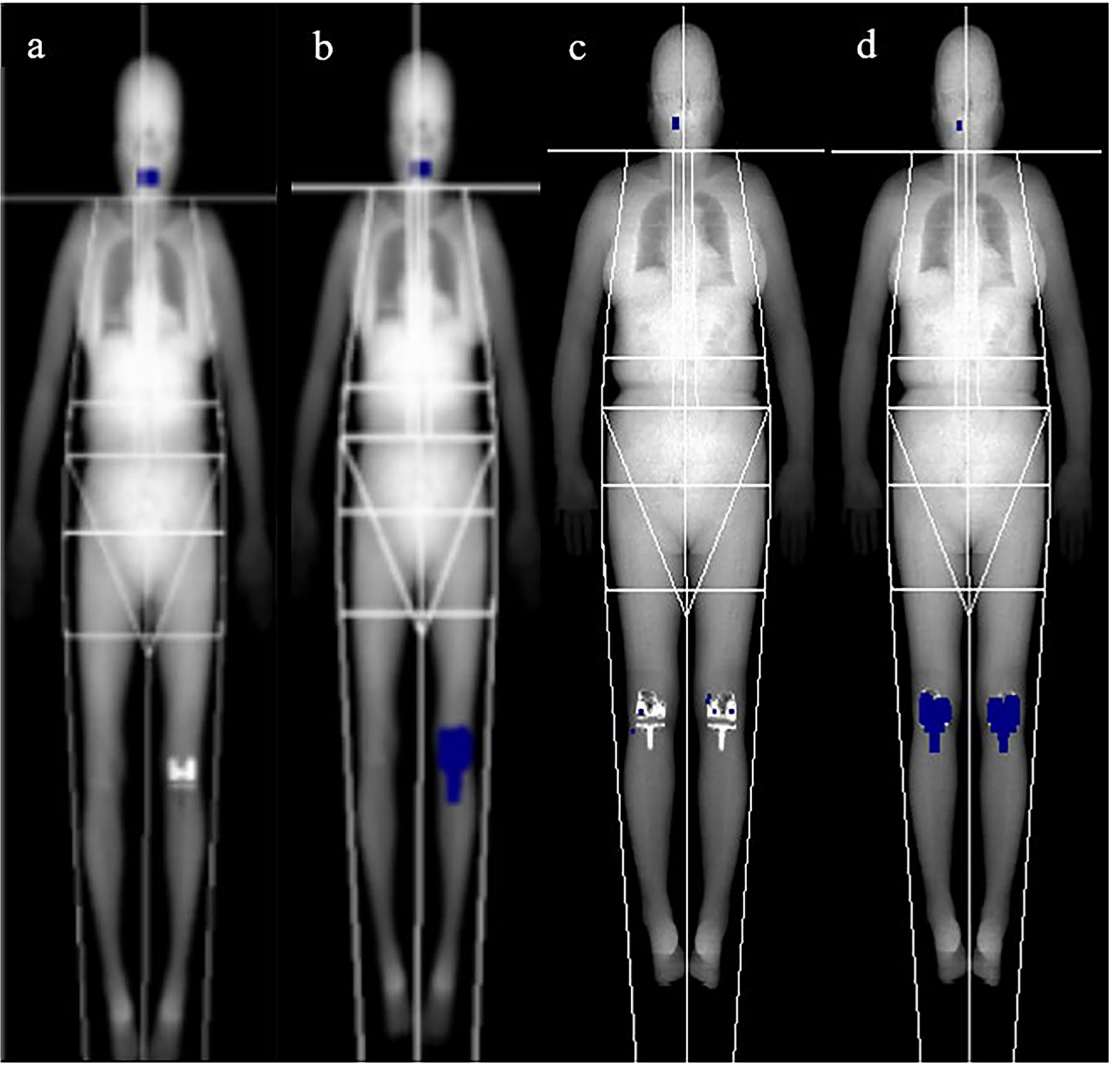
Dual X-ray absorptiometry images with and without automatic metal detection (AMD). (**a**) Left knee arthroplasty without AMD processing. (**b**) Left knee arthroplasty with AMD processing. (**c**) Bilateral knee arthroplasty without AMD processing. (**d**) Bilateral knee arthroplasty with AMD processing. *Images obtained from the Korean Frailty and Aging Cohort Study.

To control the effect of TKR on body composition measurements using DXA, AMD and a correction were performed, according to the manufacturer’s protocols. AMD is a tool included in the GE Healthcare Lunar enCORE™ software to detect metal implants and automatically remove them for analysis. Subsequently, the DXA technician manually removed the remaining pixels of the metal implants that were not removed by AMD as per the user manual. Figure 1 shows the DXA images with and without AMD processing and manual correction. For the test–retest reliability of the body composition analysis of the DXA scan, 10 participants were randomly selected among the 24 participants: three underwent right-leg TKR surgery, three underwent left-leg TKR surgery on the left leg, and four underwent TKR surgery in both legs.

### Substitution protocol using body composition of the other leg without metal implant

As Di Monaco suggested, we estimated the LM of the leg that underwent TKR by substituting it with the LM of the non-TKR leg^9^.

### Statistical analysis

The Shapiro–Wilk test was used to analyze the normal distribution of the data. Differences in values with and without AMD were analyzed using the Wilcoxon signed-rank test if variables were not normally distributed, and a paired t-test was used if variables were normally distributed. The intraclass correlation coefficient (ICC) was used by one rater to evaluate the reliability of the DXA analysis. ICC estimates and 95% confidence intervals were calculated based on a single measurement, absolute agreement, and two-way mixed model. ICC values of < 0.50 were interpreted as poor, 0.50–0.75 as moderate, 0.75–0.90 as good, and > 0.90 as excellent^24^. Fisher’s exact test or Cochran’s Q test was used to test the significance of the categorical data. The differences between values with and without AMD processing and substitution protocol were analysed using the Friedman test. All the statistical analyses were performed using SPSS software (ver. 26.0; SPSS Inc., Chicago, IL, USA).

## Results

### Characteristics of the participants

The characteristics of the participants are shown in Table 1. There were 2 men and 22 women. The mean age was 76.4 years, and the average body mass index was 26.6 kg/m^2^. Among the 24 participants, 17 were highly educated, 18 had hypertension, and nine were married.

**Table 1:** Characteristics of the study participants who underwent total knee replacement surgeries.

	Overall (n = 24)	Men (n = 2)	Women (n = 22)
Age (years)	76.4 ± 4.0	74.5 ± 3.6	76.7 ± 4.1 (70.1–84.1)
Height (cm)	153.2 ± 6.3	169.3 ± 1.2	151.7 ± 4.0 (143.7–161.0)
Weight (kg)	62.5 ± 9.0	63.5 ± 7.6 (58.0–68.8)	62.4 ± 9.3 (45.2–78.7)
Body mass index (kg/m^2^)	26.6 ± 3.3	22.2 ± 3.0 (20.1–24.3)	27.0 ± 3.1 (21.9–32.7)
Married (%)	9 (37.5%)	2 (100%)	7 (31.8%)
School education of < 7 years (%)	17 (70.8%)	0 (0.0%)	17 (77.3%)
Alcohol drinking (%)	4 (16.7%)	2 (100%)	2 (9.1%)
Current smoker (%)	0 (0.0%)	0 (0.0%)	0 (0.0%)
Hypertension (%)	18 (75.0%)	1 (50.0%)	17 (77.3%)

Values are presented as means ± standard deviations (range) or numbers (%).

### Whole-body lean mass measurements with and without AMD processing

Table 2 shows the DXA results obtained with and without AMD processing. The whole-body LM measured without AMD processing was significantly more overestimated than that measured with AMD processing. The mean whole-body LMs with and without AMD processing were 36,953 g and 36,259 g, respectively; LM without AMD processing was overestimated by 1.9% compared with that with AMD processing. The BMC and FM without AMD processing were also overestimated by 16.2% and 4.3%, respectively, compared with those with AMD processing. The intrapersonal test–retest reliabilities of the whole-body LM measurements with and without AMD processing were 0.994 and 0.946, respectively (Table S2). Additionally, total LM with AMD processing seemed to show a stronger association with grip strength than without AMD processing (with AMD *r*_*s*_ = 0.564, p < 0.001; without AMD, *r*_*s*_ = 0.525, p = 0.008). However, the physical performances including SPPB, gait speed, and five-time chair stand test were not significantly associated whether with AMD processing or not (all p > 0.005) (Table S3).

**Table 2:** Body compositions of the study participants measured using whole-body dual X-ray absorptiometry with and without automatic metal detection processing (*n* = 24).

Variables	Without AMD processing	With AMD processing	*p*-value
Whole body			
BMC (g)	1764 ± 70	1553 ± 71	< 0.001
Total FM (g)	23,226 ± 6980	22,430 ± 6850	< 0.001
Total LM (g)	36,953 ± 4612	36,259 ± 4570	< 0.001
Segmental body			
Right leg FM (g)^a^	3647.4 ± 283.8	3139.1 ± 261.5	< 0.001
Left leg FM (g)^b^	3454.1 ± 321.9	2740.0 ± 216.9	< 0.001
Right leg LM (g)^a^	6017.1 ± 199.3	5493.7 ± 171.3	< 0.001
Left leg LM (g)^b^	5657.1 ± 220.1	5173.7 ± 201.8	< 0.001
Right leg BMC (g)^a^	401.3 ± 15.9	263.4 ± 14.1	< 0.001
Left leg BMC (g)^b^	377.3 ± 16.5	249.9 ± 12.1	< 0.001
SMI (kg/m^2^)	6.5 ± 0.6	6.1 ± 0.6	< 0.001

Values are presented as means ± standard deviations.

*AMD* automatic metal detection, *BMC* bone mineral content, *FM* fat mass, *LM* lean mass, *SMI* skeletal muscle mass index.

Out of the 24 participants who underwent total knee replacement (TKR) surgery, six underwent right-knee TKR surgery, four underwent left-knee TKR surgery, and 14 underwent TKR surgery in both knees.

^a^The number of participants who underwent right-knee TKR surgery was 20.

^b^The number of participants who underwent left-knee TKR surgery was 18.

### Segmental lean mass measurements with and without AMD processing

The LM, FM, and BMC of each leg measured using DXA are presented in Table 2. Twenty participants had metal implants in the right leg, among them 14 had in both legs, and 18 participants had metal implants in the left leg, among them 14 had in both legs. The LM of the right leg without and with AMD processing in 20 participants who had underwent TKR surgery on the right was 6017.1 ± 199.3 g and 5493.7 ± 171.3 g, respectively. The LM of the left leg without and with AMD processing in 18 participants who had underwent TKR surgery on the left was 5657.1 ± 220.1 g and 5173.7 ± 201.8 g, respectively (all, *p* < 0.001). The absolute difference between the LM before and after AMD processing in the right and left legs was 523.5 g and 483.4 g, respectively, and the percentage difference was 8.6% for both legs. The SMI without and with AMD processing was 6.5 ± 0.6 kg/m^2^ and 6.1 ± 0.6 kg/m^2^, respectively, and the difference was statistically significant (*p* < 0.001). Moreover, SMI with AMD processing (*r*_*s*_ = 0.557, p = 0.005) seemed to show a stronger association with grip strength than without AMD processing (*r*_*s*_ = 0.482, p = 0.017). Nevertheless, SPPB, gait speed, and five-time chair stand test were not significantly associated with either with and without AMD processing (all, p > 0.005) (Table S3). The intrapersonal test–retest reliability of the LM measurements without AMD processing was 0.955 and 0.960 in the right and left legs, respectively. The intrapersonal test–retest reliability of the LM measurements with AMD processing was 0.999 in both legs (Table S4).

### Lean mass measurement according to substitution protocol application in the 10 participants who underwent single-leg TKR surgery

Table 3 shows comparisons of the whole-body and segmental DXA measurements without and with AMD processing and substitution protocol application in participants with single-leg TKRs (n = 10). Whole-body LM calculated using the substitution protocol was significantly lower than that without AMD processing (*p* = 0.008), but not significantly higher than that with AMD processing (*p* = 0.074). LM measurement using substitution protocol of the leg that had underwent TKR surgery was significantly lower than that without AMD processing (*p* = 0.005) and significantly higher than that with AMD processing (*p* = 0.013).

**Table 3:** Body compositions measured using whole-body dual X-ray absorptiometry according to automatic metal detection processing and application of substitution protocol among 10 study participants who underwent unilateral total knee replacement only.

	Without AMD processing	With AMD processing	Substitution protocol applied	*p-*value
Whole body				
Total BMC (g)	1769.3 ± 371.0	1649.7 ± 379.0^a^	1697.9 ± 393.0^a, b^	< 0.001
Total FM (g)	23,083.4 ± 8350.0	22,559.5 ± 8199.0^a^	22,768.4 ± 8291.0	0.001
Total LM (g)	35,936.4 ± 4682.0	35,618.8 ± 4950.0	35,654.0 ± 4649.0^a^	0.008
Segmental body				
Leg that underwent TKR surgery, FM (g)	3556.0 ± 1521.0	3036.8 ± 1377.0^a^	3213.1 ± 1441.0^a^	0.001
Leg that underwent TKR surgery, LM (g)	5699.3 ± 1066.0	5185.4 ± 936.0^a^	5416.9 ± 1015.0^a, b^	0.045
Leg that underwent TKR surgery, BMC (g)	377.3 ± 64.0	260.1 ± 73.0^a^	305.9 ± 82.0^a, b^	< 0.001
SMI (kg/m^2^)	6.31 ± 0.71	6.10 ± 0.69^a^	6.19 ± 0.69^a,b^	< 0.001

Substitution protocol: substituting the LM of the leg that underwent TKR with that of the leg that did not undergo TKR.

*AMD* automatic metal detection, *BMC* bone mineral content, *FM* fat mass, *LM* lean mass, *TKR* total knee replacement, *SMI* skeletal muscle mass index.

^a^Statistically significant difference between results without and with AMD processing and between results with AMD processing and substitution protocol application.

^b^Statistically significant difference between results with AMD processing and substitution protocol application.

### Prevalence of low lean mass

**Figure 2:**
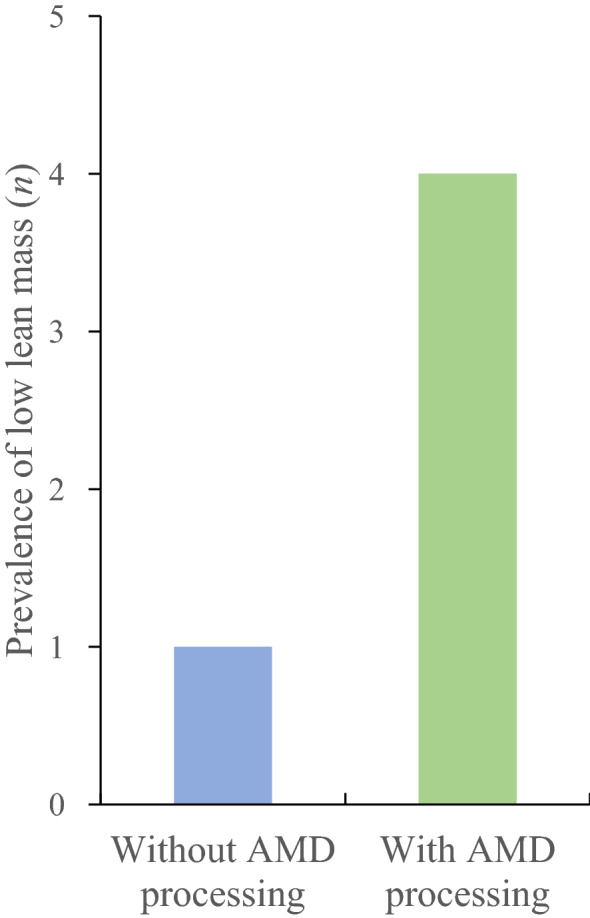
The number of participants with low lean mass (without and with AMD processing) according to the AWGS 2019 guidelines (men, < 7.0 kg/m^2^; women, < 5.4 kg/m^2^) among the 24 participants who underwent total knee replacement surgery in at least one leg. *AMD* automatic metal detection, *AWGS* Asian Working Group for Sarcopenia.

**Figure 3:**
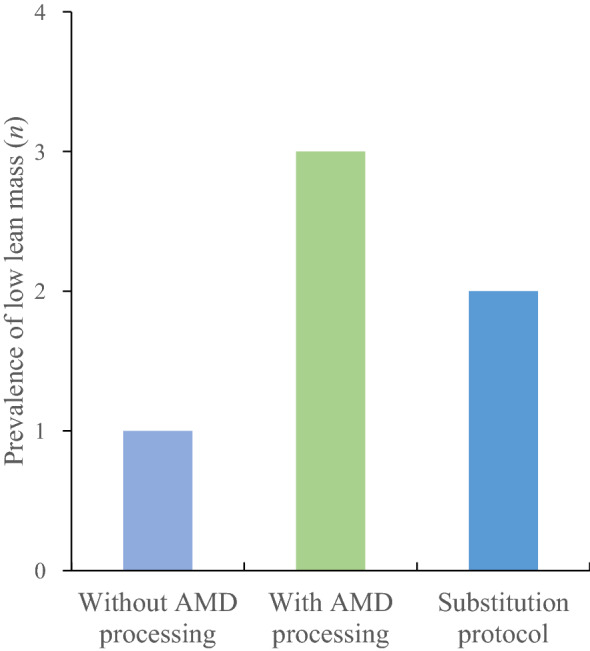
The number of participants with low lean mass (without and with AMD processing) according to the AWGS 2019 guidelines (men, < 7.0 kg/m^2^; women, < 5.4 kg/m^2^) among the 10 participants who underwent unilateral total knee replacement surgery. *AMD* automatic metal detection, *AWGS* Asian Working Group for Sarcopenia.

Figure 2 shows the number of participants who had low LM according to the AWGS 2019 guidelines (men, < 7.0 kg/m^2^; women, < 5.4 kg/m^2^) without and with AMD processing. The number of participants categorized as having low LM was only one (4.2%) without AMD processing and this came to four (16.7%) with AMD processing, respectively. Figure 3 shows the number of participants with low LM without and with AMD processing and after applying the substitution protocol among the 10 who underwent single-leg TKR surgery. The number of participants categorized as having low LM was one (10.0%), three (30.0%), and two (20.0%) without AMD processing, with AMD processing, and after applying the substitution protocol, respectively.

## Discussion

This study assessed the influence of TKR surgery on LM measurements using DXA in older adults. We found that AMD processing and substitution protocol application resulted in significantly different leg LMs in individuals who underwent single-leg TKR surgery. The LM measured using DXA with AMD processing was higher than that without AMD processing, and the LM after substitution protocol application was somewhere between these values. Furthermore, our study indicated that the number of older adults categorized as having low LM among those who underwent TKR surgery could be different depending on whether AMD processing or a substitution protocol is applied.

DXA devices measure body composition by transmitting two different energy levels of X-rays; high-density tissues (such as the bone) attenuate more energy than low-density tissues (such as soft tissues). Depending on the components of the anatomical structures and tissues, attenuation levels distinguish body composition into lean tissue, fat tissue, and bone^25^. This principle of DXA allows metal implants to overestimate body composition, as Giangregorio et al. showed that 100.6 g of exogenous metal rod increased whole body LM by 0.7% in body composition analysis using DXA compared with that without metal implants in seven participants aged > 18 years^6^. Another study also reported that in 313 older women, the LMs of the fractured legs with implants were significantly higher than those of the unfractured legs by 451.0 g when measured using DXA^22^. Similar to the results of those studies, our study found a significant overestimation of leg LM measured with DXA in individuals who had underwent TKR surgery.

Low LM measured using DXA is one of the criteria for sarcopenia^2–5^; however, there is no gold standard for measuring LM in individuals with metal implants^7^. Madsen et al. adjusted the overestimation of 7.5% in total body LM and 5.2% in leg LM in 21 older adults who had endogenous hip prosthesis using high-density detection (HDD), which is similar to AMD^8^. Similarly, our study adjusted for the overestimation of LM using AMD processing for individuals who had underwent single-leg TKR surgery, and the SMIs were significantly different between with and without AMD processing. Although not statistically significant, the number of participants who had low LM increased when AMD processing was used. Furthermore, low muscle strength is an important component in sarcopenia diagnosis. Seen in Supplementary Table S3, both whole-body LM and SMI with AMD processing had higher correlations with grip strength compared to those without AMD processing. These results are consistent with previous findings^26–28^ that skeletal muscle mass is highly correlated with grip strength. Since low muscle strength is most important for diagnosing sarcopenia^2,3^ this result may support the necessity of AMD processing in sarcopenia diagnosis for individuals with TKR implants. Therefore, the adjustment of high-density objects using software such as AMD is worth considering for LM measurements.

We found that leg LM assessment using the substitution protocol was lower than that measured without AMD processing and higher than that measured with AMD processing. LM of the leg that underwent TKR surgery is known to be lower than that of the non-operated leg. Kim et al. also reported that the volume of the vastus lateralis measured with SPECT-CT in the leg that underwent TKR surgery decreased in the operated knee and was lower than that in the unoperated leg^29^. Osteoarthritis, frequent cause of TKR surgery, might lead to decreased vastus medialis muscle volume because of insufficient use of the knee. Considering these together, LM assessed by applying the substitution protocol might still overestimate LM in patients who have TKR implants.

This study had several limitations. First, there is a lack of diversity in the DXA models and software. Our study measured body composition using GE's lunar iDXA model. Hologic and Norland also have their own DXA models and software, which may show different results^8,30^. Previous studies have also shown that measurement of body composition using DXA may be influenced by the manufacturer’s algorithm^31,32^. Norland’s HDD underestimated LM in individuals who had underwent total hip arthroplasty^8^. In addition, the software version can cause significant differences in the measurement of body composition even when the same DXA model is used^33^. Second, it may be difficult to generalize the results of this study owing to the small sample size and more extended studies are needed.

Our study showed that LM assessment in individuals with metal implants after TKR surgery could be significantly different according to the use of AMD software and substitution protocol. ALM measurement was overestimated without AMD processing compared with that with AMD processing, and this led to a decreased number of participants with low LM. Although the difference in the number of participants categorized as having low LM was not statistically significant due to the small sample size, it will make a big difference with a clinically significant meaning when LM is measured with DXA for a larger study population. Therefore, considering the effects of metal implants on sarcopenia diagnosis in individuals with metal implants in their legs is warranted.

## Supplementary Information

Supplementary Tables.

## Data Availability

The data that support the findings of this study are available on request from the corresponding author. The data are not publicly available due to privacy or ethical restrictions.
