# Supplementary material for: Effect of total knee replacement on skeletal muscle mass measurements using dual energy X-ray absorptiometry
Source: Sci Rep. 2023 Feb 19;13:2908. doi: 10.1038/s41598-023-29069-y (PMC9939411; doi:10.1038/s41598-023-29069-y)
Supplement: Supplementary file 1 — Supplementary Tables. [file 41598_2023_29069_MOESM1_ESM.pdf]

Effect of total knee replacement on skeletal muscle mass measurements using dual energy X-ray absorptiometry

Authors' names and institutions:

Jae Young Jang [1], Miji Kim [2*], Daehyun Lee [1], Chang Won Won [3*]

[1] Department of Biomedical Science and Technology, Graduate School, Kyung Hee University, Seoul 02447, Korea

[2] Department of Biomedical Science and Technology, College of Medicine, East-West Medical Research Institute, Kyung Hee University, Seoul 02447, Korea

[3] Elderly Frailty Research Center, Department of Family Medicine, College of Medicine, Kyung Hee University, Seoul 02447, Korea

* These authors contributed equally to the manuscript.

Authors' E-mail:

Jae Young Jang (jyjang026@gmail.com), Miji Kim (mijiak@khu.ac.kr), Daehyun Lee (lyh737@naver.com), Chang Won Won (chunwon62@naver.com)

Corresponding authors: These authors equally contributed to this work as co-corresponding authors.

Miji Kim, PhD

Department of Biomedical Science and Technology, College of Medicine, East-West Medical Research Institute, Kyung Hee University, Seoul, 02447, Korea

Tel: +82-2-958-2840; Fax: +82-2-958-2836; E-mail: mijiak@khu.ac.kr

Chang Won Won, MD, PhD

Department of Family Medicine, College of Medicine, Kyung Hee University, 23, Kyung Hee Dae-ro, Dongdaemun-gu, Seoul, 02447, Korea

Tel: +82-2-958-8700; Fax: +82-2-958-8699; E-mail: chunwon62@naver.com

Online supporting material

Supplementary Table S1. Original research articles that used dual X-ray absorptiometry (DXA) to measure lean mass (n=595) according to considerations of the presence of metal implants, DXA device manufacturer, and DXA device model.

Supplementary Table S2. Differences between body composition measurements before and after automatic metal detection processing (*n*=24).

Supplementary Table S3. Correlation of muscle function indicators with body compositions between with and without AMD processing.

Supplementary Table S4. Intraclass correlation between test and retest of dual energy X-ray absorptiometry measurements according to automatic metal detection processing (*n*=10).

Supplementary Table S1. Original research articles that used dual X-ray absorptiometry (DXA) to measure lean mass (n=595) according to considerations of the presence of metal implants, DXA device manufacturer, and DXA device model.

**Exclusion of participants with metal prosthesis**	**Manufacturer**	**Dual X-ray absorptiometry model**					
Not Mentioned (517)	GE	Lunar (18)	iDXA (50)	DPX (41)	Prodigy (77)	NS (3)	
	Hologic	QDR (111)	Discovery (102)	Explorer (5)	Horizon (8)	Delphi (14)	NS (15)
	Medic	DR C 12 (3)					
	Norland	Mark 2 (1)					
	Stratos	DR (3)					
	Multiple manufacturers	(19)					
	Not Mentioned	(56)					
Mentioned (78)	GE	only Lunar (5)	iDXA (5)	DPX (9)	Prodigy (13)		
	Hologic	QDR (17)	Discovery (11)	Horizon (1)	Delphi (5)	NS (2)	
	Norland	Excell plus (1)	XR 36 (1)				
	Multiple manufacturers	(5)					
	Not mentioned	(3)					

Abbreviations: GE, General Electric; NS, not stated.

The following keywords were used to search for studies published from January 2010 to August 2021: “sarcopenia” AND “dual energy x-ray absorptiometry.”

Supplementary Table S2. Differences between body composition measurements before and after automatic metal detection processing (n=24).

Variables	Absolute difference (g)		Percentage difference (%)	
	**Mean**	**Standard deviation**	**Mean**	**Standard deviation**
Whole body				
BMC (g)	211.2	102.4	13.3	0.07
FM (g)	769.8	343.4	3.6	1.6
LM (g)	694.5	503.5	1.9	1.2
Segmental body				
Right leg FM (g)[a]	508.25	156.559	14.6	0.04
Left leg FM (g)[b]	714.11	991.041	18.2	0.13
Right leg LM (g)[a]	523.45	212.686	8.6	0.03
Left leg LM (g)[b]	483.39	177.720	8.6	0.03
Right leg BMC (g)[a]	137.90	38.815	34.6	0.08
Left leg BMC (g)[b]	127.39	45.373	33.9	0.1
SMI (kg/m^2)	0.336	0.153	5.3	2.3

[a] The number of participants who underwent right-knee TKR surgery was 20.

[b] The number of participants who underwent left-knee TKR surgery was 18.

Abbreviations: SMI, appendicular lean mass index; AMD, automatic metal detection; BMC, bone mineral content; FM, fat mass; LM, lean mass.

Supplementary Table S3. Correlation of muscle function indicators with body compositions between with and without AMD processing.

	Grip strength				Gait speed				SPPB				Five-time chair stand test			
	Without AMD processing		With AMD processing		Without AMD processing		With AMD processing		Without AMD processing		With AMD processing		Without AMD processing		With AMD processing	
	r_s	*p*-value	r_s	*p*-value	r_s	*p*-value	r_s	*p*-value	r_s	*p*-value	r_s	*p*-value	r_s	*p*-value	r_s	*p*-value
Whole body																
BMC (g)	0.220	0.302	0.246	0.246	0.086	0.689	-0.030	0.891	-0.037	0.865	-0.116	0.588	0.163	0.448	0.130	0.544
Total FM (g)	-0.322	0.125	-0.313	0.136	-0.243	0.253	-0.277	0.190	-0.147	0.494	-0.140	0.516	-0.013	0.952	-0.014	0.949
Total LM (g)	0.525	0.008*	0.564	<0.001**	0.044	0.837	0.050	0.815	-0.137	0.524	-0.154	0.473	0.240	0.259	0.273	0.197
Segmental body																
Right leg FM (g)[a]	-0.406	0.076	-0.380	0.098	-0.325	0.162	-0.373	0.105	0.020	0.934	0.006	0.981	-0.011	0.965	-0.005	0.985
Left leg FM (g)[b]	-0.317	0.200	-0.261	0.295	-0.167	0.507	-0.264	0.289	-0.125	0.622	-0.076	0.765	-0.236	0.345	-0.236	0.345
Right leg LM (g)[a]	0.259	0.013*	0.308	0.186	-0.015	0.950	0.008	0.975	-0.333	0.152	-0.309	0.185	0.171	0.470	0.122	0.609
Left leg LM (g)[b]	0.303	0.222	0.379	0.121	-0.214	0.394	-0.218	0.385	-0.170	0.501	-0.097	0.702	0.114	0.654	0.026	0.919
Right leg BMC (g)[a]	0.245	0.298	0.445	0.049	-0.113	0.635	0.071	0.767	-0.629	0.003	-0.200	0.399	0.463	0.040	0.353	0.126
Left leg BMC (g)[b]	0.020	0.937	0.337	0.171	-0.218	0.384	-0.389	0.110	-0.336	0.172	-0.278	0.264	0.289	0.246	0.121	0.633
SMI (kg/m^2)	0.482	0.017*	0.557	0.005*	0.146	0.497	0.053	0.805	-0.150	0.486	-0.168	0.432	0.140	0.514	0.120	0.576

Abbreviations: SMI, appendicular lean mass index; AMD, automatic metal detection; BMC, bone mineral content; FM, fat mass; LM, lean mass

Supplementary Table S4. Intraclass correlation between test and retest of dual energy X-ray absorptiometry measurements according to automatic metal detection processing (*n*=10).

	Without AMD processing		ICC	With AMD processing		ICC
	Test	Retest		Test	Retest	
Whole body						
LM (g)	37,973 ± 6,746	36,938 ± 5,941	0.946	37,228 ± 6,371	36,472 ± 6,072	0.994
SMI (kg/m^2)	6.42 ± 0.81	6.18 ± 0.70	0.960	6.11 ± 0.72	6.11 ± 0.69	0.999
Segmental body						
Right leg LM (g)	5,887 ± 1,345	5,539 ± 1,025	0.955	5,475 ± 996	5,448 ± 993	0.999
Left leg LM (g)	5,839 ± 1,235	5,546 ± 1,167	0.971	5,482 ± 1,238	5,466 ± 1,193	0.999

ICC values of <0.50 were interpreted as poor, 0.50–0.75 as moderate, 0.75–0.90 as good, and >0.90 as excellent.
Abbreviations: SMI, appendicular lean mass index; AMD, automatic metal detection; ICC, intraclass correlation coefficient; LM, lean mass.
